# Vestibulo-sympathetic Reflex and Pupillary Responses Elicited by Vertical Semicircular Canal Stimulation

**DOI:** 10.21203/rs.3.rs-10833093/v1

**Published:** 2026-09-30

**Authors:** Fumihiro Mochizuki, Yoshiyuki Sasano, Alexander Kiderman, Bernat Miro, Madison Hawthorne, Michael Hoffer, Erin Williams

**Affiliations:** University of Miami Miller School of Medicine; University of Miami Miller School of Medicine; Spryson America, Inc; University of Miami Miller School of Medicine; University of Miami Miller School of Medicine; University of Miami Miller School of Medicine; University of Miami Miller School of Medicine

**Keywords:** vestibulo-sympathetic reflex, vertical semicircular canals, spectral pupillometry, rotary chair test, phase-locking, velocity-referenced gain

## Abstract

The vestibulo-sympathetic reflex has been studied mainly using horizontal semicircular canal and otolith stimulation, whereas the autonomic role of the vertical canals remains unclear. We quantified cardiovascular and pupillary responses to vertical canal stimulation and compared stimulus-frequency-locked pupil oscillations between vertical and horizontal rotations in 18 healthy adults. Vertical stimulation used a 70°-tilted rotary chair aligning the vertical canal planes with the earth-horizontal axis. Participants underwent sinusoidal harmonic acceleration at 0.08, 0.16, and 0.64 Hz and the vertical velocity step test at 100°/s. Systolic blood pressure rose 5.8–7.8 mmHg with medium-to-large effect sizes but did not survive multiple-comparison correction. Pupil area increased significantly at all frequencies, and vertical stimulation produced larger stimulus-frequency-locked oscillation amplitude compared to horizontal stimulation (β = 0.568 mm^2^, p = 0.018). Additionally, the pupillary oscillation was phase-locked to the stimulus per canal plane, reaching significance at 0.64 Hz (sign-aligned cross-plane phase-locking value 0.27–0.37; Rayleigh p = 0.015). A two-stage gain model (cupula velocity transduction filtered by iris smooth-muscle dynamics; gain ≈ 0.09 mm^2^/(deg/s); iris corner ≈ 0.71 Hz) was more consistent with a velocity-coded than an acceleration-coded drive, although the sample size precluded resolving the absolute phase. These findings suggest that vertical canal inputs may engage human vestibulo-autonomic pathways and that spectral pupillometry may provide a noninvasive index of vestibulo-sympathetic reflex function.

## 1. INTRODUCTION

The vestibular system serves a dual role in human physiology: it stabilizes gaze and posture during head movement, and it coordinates rapid autonomic adjustments that maintain cardiovascular homeostasis during postural transitions. This latter function – the vestibulo-autonomic system – constitutes a critical neural network linking vestibular sensory input with sympathetic, parasympathetic, and respiratory regulation [1–3]. Its clinical importance is underscored by the high prevalence of orthostatic intolerance, syncope, and autonomic dysregulation in patients with peripheral and central vestibular disorders [4], yet the specific contribution of individual vestibular end-organs to autonomic control remains incompletely understood.

The neural architecture of this system has been delineated through both animal electrophysiology and human functional studies. Primary afferents from the vestibular organs synapse in the vestibular nuclei (VN), from which descending projections reach the rostral ventrolateral medulla (RVLM) – the principal “pressor center” providing tonic excitatory drive to preganglionic sympathetic neurons in the intermediolateral cell column (IML) [4–6]. Holstein et al. [7] demonstrated direct projections from the caudal vestibular nuclei to the ventrolateral medulla in rats, confirming the anatomical substrate for this pathway. This VN-to-RVLM pathway constitutes the vestibulo-sympathetic reflex (VSR), which rapidly buffers arterial pressure changes during head and body movement (Fig. 1). A distinguishing feature of the VSR is its capacity for feedforward control: unlike baroreflexes, which respond reactively to blood pressure changes, the VSR can be engaged before significant hemodynamic shifts occur, providing anticipatory cardiovascular adjustments during postural transitions [6]. Furthermore, VSR gain is not fixed: responses are exaggerated in decerebrate animal preparations, indicating that higher brain centers, including the posterior cerebellar vermis, calibrate and dampen vestibulo-sympathetic outflow [6].

Previous studies of the VSR have focused predominantly on horizontal semicircular canal (SCC) and otolith contributions. Direct recordings of muscle sympathetic nerve activity (MSNA) have established that otolith-driven stimuli – including head-down neck flexion, off-vertical axis rotation (OVAR), and linear accelerations – reliably modulate sympathetic outflow in healthy subjects [6, 8, 9]. In contrast, findings from horizontal canal paradigms have been inconsistent: Costa et al. [10] and Carter and Ray [8] found no autonomic changes during caloric stimulation or passive horizontal head rotation, while Kaufmann et al. [11] demonstrated that rotation-synchronous MSNA peaks were present during OVAR but absent during earth-vertical axis rotation (EVAR) [8, 10, 11]. Together, these findings indicate that otolithic rather than horizontal canal inputs likely dominate the VSR in humans [6, 8], and that vestibular and baroreflex contributions to sympathetic control are additive rather than redundant [6, 8, 12]. Consistent with these negative horizontal canal findings, Yang et al. [13] found no direction-dependent sympathetic effects on pupil diameter during whole-body yaw rotation [13]. Notably, direct MSNA recordings during horizontal canal stimulation in humans have shown no measurable sympathetic modulation [14], and recent reviews confirm that otolith organs remain the dominant peripheral driver of vestibulosympathetic outflow [15].

The autonomic consequences of vertical SCC stimulation, however, remain largely unexplored. The vertical canals are activated during pitch and roll movements – precisely the conditions in which rapid autonomic compensation is most needed. In animal studies, vestibular stimulation produces regionally patterned sympathetic changes across different vascular beds [6], and Rice et al. [16] recently demonstrated that pulsed infrared stimulation of a single vertical SCC is sufficient to evoke cardiovascular changes [6, 16]. Advances in rotational chair technology now enable selective vertical canal stimulation in humans [17, 18], creating an opportunity to characterize this pathway [17, 18].

The pupil provides a unique window into vestibulo-autonomic function. Pupil diameter reflects the balance between sympathetic innervation of the dilator pupillae (via the superior cervical ganglion) and parasympathetic innervation of the sphincter pupillae (via the Edinger-Westphal nucleus and ciliary ganglion). Kitajima and colleagues [19, 20] demonstrated that auditory-pupillary responses are partly mediated by otolith-autonomic pathways and are altered in vestibular neuritis [19, 20]. Recent clinical studies have further linked otolith dysfunction to autonomic dysregulation in orthostatic dizziness [21] and POTS [21, 22]. However, to our knowledge, no prior study has examined whether the pupil exhibits stimulus-frequency-locked oscillation during controlled vestibular stimulation, or whether such oscillation is canal-plane specific.

We employed two complementary paradigms – the vertical sinusoidal harmonic acceleration test (vSHA) and the vertical velocity step test (vVST) – to quantify cardiovascular and pupillary responses to selective vertical SCC stimulation. Horizontal SHA at the same frequencies served as a within-study control. Beyond mean pupil area changes, we performed spectral analysis to quantify phase-locking of the pupillary oscillation to the stimulus and fitted a two-stage gain model (cupula velocity transduction filtered by iris smooth-muscle dynamics) to characterize the vestibulo-pupillary pathway. We hypothesized that: (1) vertical canal stimulation would produce greater pupil area modulation at the stimulus frequency than horizontal stimulation, and (2) the pupillary oscillation would be phase-locked to the stimulus in a canal-plane-specific manner, with a velocity-referenced gain profile consistent with cupula velocity transduction.

## 2. RESULTS

### 2.1. Participant Characteristics

All 18 participants completed vSHA and horizontal SHA (hSHA) testing. Of these, 16–17 had usable vVST autonomic data. No participant experienced nausea or vomiting.

### 2.2. Autonomic Responses

#### 2.2.1. vSHA

Blood pressure increased consistently from baseline across all three frequencies, with medium-to-large effect sizes for systolic blood pressure (SBP; r = 0.52–0.61), diastolic blood pressure (DBP; r = 0.53–0.60), and mean arterial pressure (MAP; r = 0.50–0.58) (Table 1). The largest effects were observed at 0.16 Hz (SBP: +7.79 ± 12.49 mmHg, W = 138, p-value = 0.024, r = 0.61). After Benjamini-Hochberg (BH) correction across 15 tests, no individual comparison reached the adjusted significance threshold (all p-adjusted > 0.05), consistent with the exploratory sample size (n = 18). Heart rate (HR) and respiratory rate (RR) did not change appreciably at any frequency.

#### 2.2.2. vVST

As seen in Table 2, blood pressure increases during vVST were larger than during vSHA, with large effect sizes for SBP (r = 0.65–0.68), DBP (r = 0.63–0.68), and MAP (r = 0.62–0.63). Combined across positions (n = 17), SBP increased by 8.74 ± 11.38 mmHg (W = 127, p-value = 0.018, r = 0.66). After BH correction across 15 tests, blood pressure metrics approached but did not reach the adjusted significance threshold (all p-adjusted = 0.053). HR showed a medium effect size in the direction of increase (+ 2.26 ± 5.23 bpm, r = 0.45, p-adjusted = 0.146). RR did not change. No differences were observed between the left anterior–right posterior (LARP) and right anterior–left posterior (RALP) canal plane (LARP n = 16, RALP n = 16), with comparable effect sizes (SBP: r = 0.68 and 0.65; DBP: r = 0.68 and 0.65; MAP: r = 0.62 and 0.62, respectively).

### 2.3. Pupillary Responses

#### 2.3.1. Tonic Responses

Pupil area increased significantly from baseline during vSHA at all three frequencies (p < 0.001), with mean dilation of 14–18 mm^2^. Baseline pupil area was smaller during vertical testing (72.2 mm^2^) than horizontal testing (78.9 mm^2^; paired t(16)=−3.85, p = 0.001), likely reflecting altered gravitational loading in the tilted position. This tonic difference does not affect the spectral analysis, which extracts oscillatory amplitude after linear detrending.

#### 2.3.2. Spectral Pupillometry: Vertical vs. Horizontal

Representative vertical and horizontal pupil traces at 0.64 Hz are shown in Fig. 2; vertical rotation produced a visible stimulus-frequency oscillation absent during horizontal rotation. Vertical SHA produced larger pupil oscillation amplitude at the stimulus frequency than horizontal SHA (linear mixed-effects model [LME]: β=+0.568 mm^2^, SE = 0.236, t(77.9) = 2.41, p = 0.018). The Condition × Frequency interaction was not significant (0.16 Hz: ß=−0.25, p = 0.459; 0.64 Hz: ß=−0.23, p = 0.502), indicating a consistent vertical advantage across all frequencies.

Sensitivity analyses confirmed that the effect was robust to inclusion of baseline pupil area as a covariate (β=+0.57, p = 0.020; baseline p = 0.930), that random slopes did not improve model fit (p = 0.48), and that the effect was directionally consistent when oscillation amplitude was normalized by baseline pupil area to control for Weber’s law (normalized LME: β=+0.76%, p = 0.058). Notably, the vertical condition had a smaller baseline pupil (72.2 vs. 78.9 mm^2^), so Weber’s law would predict smaller – not larger – oscillations in the vertical condition, whereas larger oscillations were observed. However, LME residuals deviated from normality (Shapiro-Wilk W = 0.84, p < 0.001), and a permutation test (5000 iterations) yielded p = 0.104. The consistent direction across all frequencies (Cohen’s d = 0.39–0.69) and convergent evidence from the velocity-referenced gain analysis provide additional support.

Post-hoc paired t-tests showed medium-to-large effect sizes at all frequencies, with the largest at 0.64 Hz (n = 15; d = 0.69; p-value = 0.018; p-adjusted = 0.054) and 0.16 Hz (n = 15; d = 0.60; p-value = 0.036; p-adjusted = 0.054). At 0.08 Hz, the effect was medium and in the same direction (n = 17; d = 0.39; p-value = 0.128). No individual frequency reached significance after BH correction, although the medium-to-large effects were directionally consistent across all frequencies (d = 0.39–0.69). The vertical-minus-horizontal difference spectrum isolated the VSR-specific signal, showing a positive peak at the stimulus frequency at all three test frequencies (Fig. 3).

The pupillary oscillation was phase-locked to the stimulus per canal plane: the sign-aligned cross-plane phase-locking value increased with frequency (0.27, 0.31, and 0.37 at 0.08, 0.16, and 0.64 Hz) and reached significance at 0.64 Hz (Rayleigh p = 0.015), with the two lower frequencies below threshold (Fig. 4). No significant asymmetry was observed between LARP and RALP. Pupil oscillation amplitude and velocity-referenced gain were also larger during vertical than horizontal SHA at all three frequencies (Table 3).

### 2.4. Velocity-Referenced Pupil Gain Model

Two competing gain models — a velocity-coded drive and an acceleration-coded drive, sharing a common iris smooth-muscle low-pass — were fitted to the velocity-referenced gain of the vertical condition at the three test frequencies. The velocity-coded model fit the gain approximately 53-fold better than the acceleration-coded alternative, identifying velocity rather than acceleration coding from the gain (Table 4; Fig. 4).

The velocity-referenced gain decreased only mildly across 0.08–0.64 Hz (0.107, 0.077, and 0.070 mm^2^/(deg/s)), the near-flat profile expected of a velocity-coded drive shaped by an iris smooth-muscle low-pass. Because the model is fitted to the gain alone, it does not estimate a neural delay, and the stimulus-frequency phase was not reliably resolved at this sample size; the velocity-coding inference therefore rests on the gain profile rather than on the absolute phase.

### 2.5. Velocity Step Pupil Time Course

Peri-stimulus analysis revealed progressive divergence between vertical and horizontal conditions after acceleration onset. During vertical steps, pupil area changed from baseline while remaining stable during horizontal steps.

In an exploratory post-rotatory analysis, the vertical-horizontal difference began to change 0.22 s after the gyro-detected chair stop (both smoothed-threshold and per-bin t-test methods converged; bootstrap 95% CI: 0.22–6.22 s, 389/1000 iterations; **Supplementary Figure S2**). This provisional ~ 0.22 s latency is the only available estimate of pathway delay, because the gain model does not estimate a delay and the stimulus-frequency phase could not be resolved at this sample size; the point estimate coincides with the lower bound of a wide bootstrap interval and fewer than half of iterations converged, so it should be read as an order-of-magnitude estimate only.

## 3. DISCUSSION

This study provides preliminary evidence suggesting that vertical SCC stimulation engages vestibule-autonomic pathways in humans. Vertical SHA produced consistent cardiovascular pressor responses with medium-to-large effect sizes, larger stimulus-frequency-locked pupil oscillation amplitude than horizontal SHA (LME p = 0.018; permutation p = 0.104) with consistent medium-to-large effects across all tested frequencies, and a pupillary oscillation that was phase-locked to the stimulus per canal plane (significant at 0.64 Hz) with a velocity-referenced gain profile consistent with velocity-coded drive. Together, these findings extend the VSR literature, which has focused predominantly on horizontal canal and otolith contributions, by providing an initial characterization of the vertical canal pathway from vestibular afferent to iris smooth muscle.

The within-study horizontal SHA control enabled direct assessment of whether the pupillary response was specific to vertical canal activation. Consistent effect sizes across frequencies (d = 0.39–0.69, all favoring vertical) and the canal-plane-specific phase-locking of the pupillary oscillation are consistent with canal-plane specificity, though the permutation result (p = 0.104) indicates that confirmatory studies are needed. This pattern is consistent with animal data showing predominantly otolith and vertical canal inputs to RVLM neurons [5], negative human MSNA findings during horizontal rotation [8, 11], and the absence of sympathetic pupil effects during yaw rotation alone [8, 11, 13]. The vertical-minus-horizontal difference spectrum further isolates the VSR-specific component by removing signals common to both conditions, including vestibular-induced arousal, chair vibration, and VOR-related measurement artifact.

To our knowledge, this is among the first human spectral pupillometry studies of the vertical-canal-driven vestibulo-pupillary pathway. The velocity-referenced gain (α ≈ 0.09 mm^2^/(deg/s) [95% CI: 0.07–0.12]) provides a preliminary quantification of overall pathway sensitivity from the cupula-coded velocity signal to pupil area, with the near-flat gain profile consistent with velocity coding. A neural delay was not estimated, because the gain model contains no delay term and the spectral phase was unresolved; the only provisional delay estimate, the ~ 0.22 s post-rotatory divergence onset, while shorter than MSNA-based estimates (0.4–0.66 s [11, 23]), is broadly consistent with conduction across the VN-RVLM-IML-superior cervical ganglion (SCG) sympathetic chain. A synaptic-chain minimum of approximately 80–100 ms nonetheless sets a lower bound on any true delay. Furthermore, the wide bootstrap interval precludes strong claims about parasympathetic versus sympathetic dominance from this dataset; resolving the contribution from each branch (i.e., the VN-to-Edinger-Westphal circuit is more direct than the full spinal sympathetic chain) will require either pharmacological dissection (atropine, phenylephrine) in pre-clinical models or denser frequency sampling. Lastly, the fitted iris-muscle corner frequency (f_m ≈ 0.71 Hz [95% CI: 0.45–1.8]) should be interpreted as a descriptive low-pass parameter; experimental studies support viscoelastic behavior of both sphincter and dilator iris smooth muscle [24]. Importantly, pupil dilation can arise from sympathetic activation of the dilator pupillae or parasympathetic withdrawal from the sphincter pupillae [25, 26]; our paradigm does not dissociate these branches, so the observed oscillations may reflect either or both autonomic limbs.

Because the absolute stimulus-frequency phase was not resolved, the velocity-coding inference rests on the gain profile rather than on the phase. Model predictions outside the tested band (e.g., the 0.32 Hz gain prediction) should accordingly be considered exploratory. This velocity-coding pattern is comparable to Yates [27], who reported splanchnic nerve responses tracking near stimulus position during pitch-plane stimulation, whereby cupula-derived velocity drives the pupillary response. Notably, by measuring the pupil rather than neural activity, we obtain a gain model that includes the iris smooth-muscle stage, providing a metric that is both mechanistically informative and clinically accessible; prior VSR characterizations based on neural recordings reflect the central pathway only and cannot recover this terminal stage [6, 27].

To facilitate future validation, our model yields an a priori velocity-referenced gain prediction at 0.32 Hz (a frequency not used in the present study) of ≈ 0.08 mm^2^/(deg/s) [95% CI: 0.06–0.11]. The discriminating test of coding is the low-frequency gain slope: velocity coding predicts a gain that is approximately flat against angular velocity, whereas acceleration coding predicts a gain that falls steeply toward low frequency. Testing at 0.32 Hz and extending down to 0.04 Hz, with a per-plane estimator and a sample powered to resolve phase-locking, would settle the encoding and recover the neural delay.

These findings should be interpreted within the framework established by Yates et al. [6], who demonstrated that otolith organs dominate the human VSR and that the reflex provides feedforward cardiovascular control. Our results extend this framework by showing that vertical canal activation engages autonomic pupillary pathways during the pitch and roll movements most relevant to blood pressure regulation. Canal and otolith contributions cannot be fully dissociated: the 70° tilt produces a sustained otolithic stimulus alongside the rotational one, and although the spectral analysis isolates the oscillatory component at the stimulus frequency, tonic otolithic input could modulate the gain of the canal-driven pathway without generating an oscillatory signal — a priming effect that would not be captured by spectral analysis and could partly explain the vertical-horizontal contrast. Notably, Skoric et al. [28] recently demonstrated that air-conducted sound stimuli engage the VSR hemodynamically, further supporting the view that multiple vestibular end-organs converge on this pathway. Critically, the 70° backward tilt required for vertical canal alignment introduces sustained otolith stimulation that may prime or modulate the observed pupillary response. Because otolith organs are the dominant driver of the human VSR [6, 11], our design cannot fully dissociate canal-specific from otolith-mediated contributions; a tilt-only control condition would help resolve this confound.

Several limitations should be considered. The primary spectral finding did not survive a nonparametric permutation test (p = 0.104), and no post-hoc frequency comparison survived BH correction; larger confirmatory samples (approximately n = 40–50 for 80% power) are needed. Additionally, the 70° tilt produced a significant reduction in tonic pupil area (72.2 vs. 78.9 mm^2^, p = 0.001), and a tilt-only control condition will be needed to fully exclude otolithic contributions. Lastly, because horizontal testing always preceded vertical testing, fatigue or arousal differences between conditions cannot be fully excluded; future studies should counterbalance condition order. Finally, no test-retest reliability assessment was performed, and establishing ICC will be important for clinical translation.

Future work should include larger confirmatory samples, tilt-only controls, pharmacological dissection of sympathetic and parasympathetic contributions, simultaneous MSNA recording during vertical canal stimulation, additional SHA frequencies to better constrain the velocity-referenced gain model, application to clinical populations including orthostatic dizziness [21], POTS [22], and vestibular syncope [29], and investigation of direction-dependent post-rotatory responses.

## 4. CONCLUSION

Vertical SCC stimulation produced pupillary modulation with consistently larger amplitude than horizontal stimulation across all tested frequencies (LME p = 0.018; permutation p = 0.104; Cohen’s d = 0.39–0.69). The pupillary oscillation was phase-locked to the stimulus per canal plane, reaching significance at 0.64 Hz (Rayleigh p = 0.015), and its velocity-referenced gain profile – near-flat and shaped by iris smooth-muscle low-pass filtering – was more consistent with velocity- than acceleration-coded drive, although the absolute phase, and thus a definitive coding determination, was not resolved at this sample size. An exploratory post-rotatory analysis yielded a provisional pathway-delay estimate (~ 0.22 s). These findings suggest that vertical canal inputs engage human vestibulo-autonomic pathways and that spectral pupillometry during standard rotary chair testing may serve as a noninvasive, clinically accessible index of VSR function.

## 5. METHODS

### 5.1. Study Participants

We enrolled 18 adults (8 males, 10 females; mean ± SD age 27.0 ± 3.2 years, range 22–34) with no history of vestibular disorders, traumatic brain injury, neurological disease, or medications known to affect autonomic function. Participants were instructed to abstain from caffeine and vigorous exercise for at least 4 hours before testing. All procedures were approved by the University of Miami Institutional Review Board (IRB# 20190034) and were performed in accordance with relevant guidelines and regulations. All participants provided written informed consent before participation.

### 5.2. Devices

#### 5.2.1 Rotational chair

Whole-body rotational testing was performed using the Neuro-Otologic Test Center (NOTC) rotary chair system supplied by Spryson America, Inc. (Pittsburgh, PA), as previously described for vertical semicircular canal testing [18]. The system includes binocular infrared video-oculography with a spatial resolution of 0.1° and a sampling rate of 250 frames/s, as well as an embedded six-degree-of-freedom inertial sensor. The chair was housed in a light-proof enclosure and modified to allow 70° backward tilt, with a customizable headrest that could be rotated ± 45° to align the LARP or RALP canal plane with the earth-horizontal plane. Participants were secured using a four-point lap and shoulder belt system, with the head and neck stabilized using molded foam supports, a forehead strap, and a polyurethane neckband.

#### 5.2.2. Noninvasive Cardiovascular Output Monitor

Beat-to-beat cardiovascular measurements were obtained using a Caretaker wireless noninvasive cardiovascular output monitor (Caretaker Medical, LLC, Charlottesville, VA). The device continuously recorded SBP, DBP, MAP, HR, and RR from the finger arterial pressure waveform at 250 Hz. Cardiovascular and video-oculography signals were time-aligned post hoc using digital timestamps.

### 5.3 Procedure

#### 5.3.1 Rotational chair battery

Rotational testing was performed as previously described for vertical semicircular canal assessment [18]. After goggle calibration with ± 10° horizontal and vertical fixation targets, horizontal SCC testing was performed with the head positioned to align the horizontal SCC plane with the earth-horizontal plane. Participants underwent horizontal SHA at 0.08, 0.16, and 0.64 Hz and horizontal VST.

For vertical SCC testing, the chair was tilted backward 70° and the headrest was rotated 45° to align the LARP or RALP canal plane with the earth-horizontal plane. Eye-velocity calibration was performed using an earth-fixed LED target approximately 1 m from the chair during sinusoidal rotation at 0.25 Hz with ± 10° amplitude. Participants then underwent vSHA at 0.08, 0.16, and 0.64 Hz and vVST in both vertical canal planes. vVST consisted of acceleration at 125°/s^2^ to 100°/s, 60 s of constant-velocity rotation, and deceleration at 125°/s^2^ to rest, repeated in both clockwise and counterclockwise directions. LARP/RALP order was randomized, and verbal alerting tasks were used throughout testing.

Throughout this manuscript, clockwise (CW) and counterclockwise (CCW) refer to the direction of chair rotation about the earth-vertical axis as viewed from above. The vVST consisted of acceleration at 125°/s^2^ from 0 to 100°/s, 60 s of constant-velocity rotation, abrupt deceleration at 125°/s^2^ to rest, and 60 s of stationary recording. This sequence was performed in both CW and CCW directions, and the order of LARP and RALP testing was randomized.

The primary excitatory semicircular canal during each acceleration and deceleration phase was assigned according to Ewald’s law, based on the direction of endolymph flow relative to each canal ampulla. In the LARP position, CW acceleration and CCW deceleration primarily excited the left anterior SCC, whereas CCW acceleration and CW deceleration primarily excited the right posterior SCC. In the RALP position, the corresponding rotations primarily excited the right anterior and left posterior SCCs. The expected nystagmus directions and canal assignments for all VST conditions are summarized in **Supplementary Table S1**, and a representative LARP vVST recording is shown in **Supplementary Figure S1**.

#### 5.3.2. Data Acquisition and Processing

During each test, left/right eye position and head angular velocity were continuously collected at a sampling rate of 250 Hz. Eye position data were inverted and digitally differentiated to calculate eye velocity, which was calculated separately for the left and right eyes. Calibration of eye position was performed prior to testing using fixation targets at known angular positions for both the horizontal and vertical planes. Horizontal and vertical eye movement was used when evaluating the horizontal and vertical SCCs, respectively. Evaluation of the vertical SCCs commenced following velocity calibration.

### 5.4. Statistical Analysis

All analyses used R version 4.4.3. Statistical significance was set at alpha = 0.05. All applicable statistical tests were two-sided unless otherwise specified.

#### 5.4.1. Cardiovascular Metrics

Mean changes from 30-second pre-stimulation baseline were computed for each autonomic metric. LARP and RALP values were averaged within each participant to yield one independent observation per subject per frequency (n = 18 for vSHA; n = 16–17 for vVST, as 1 participant had an incomplete Caretaker recording and 1 additional participant lacked data for one canal position). Paired differences from zero were assessed using the Wilcoxon signed-rank test with BH correction across 15 tests per paradigm. Effect sizes were quantified using the rank-biserial correlation r = 4W/[n(n + 1)] − 1.

#### 5.4.2. Spectral Pupillometry

Binocular pupil area was averaged, blink-removed (< 500 pixels), outlier-filtered (> 1.5 SD from trial mean), interpolated, and converted to mm2. Trials with < 50% valid samples were excluded. Stimulus-frequency pupil oscillation amplitude was taken from the single-sided amplitude spectrum (full-record FFT after linear detrending and Hanning windowing). Stimulus-frequency gain and phase were estimated by least-squares fit of a single sinusoid at the exact rotation frequency to the canal-projected angular velocity and to the pupil signal; gain is the pupil-to-velocity amplitude ratio. Phase was analyzed per canal plane (LARP and RALP are approximately antiphase) and phase consistency was quantified by the phase-locking value with a Rayleigh test. Velocity- and acceleration-coded gain models, sharing one iris low-pass, were compared by their fit to the velocity-referenced gain. Linear mixed-effects models tested fixed effects of Condition (Vertical vs. Horizontal), Frequency, and their interaction, with participant as random intercept [30]. A nonparametric permutation test (5000 iterations) was conducted as a sensitivity analysis for non-normal LME residuals. Post-hoc paired t-tests (normality confirmed by Shapiro-Wilk) used BH correction.

Chair angular velocity was reconstructed from the head-mounted three-axis gyroscope by principal-component analysis (PCA), following the canal-axis decomposition described in a companion vSHA-vST manuscript (under review), which recovers the delivered rotation rather than the chair controller’s commanded voltage signal, eliminating a ~ 28 ms drive-train delay that would otherwise inflate pupil-vs-acceleration phase by ~ 0.8°, 1.6°, and 6.4° at 0.08, 0.16, and 0.64 Hz respectively. Across 196 SHA recordings, PC1 captured a mean of 99.8% of the gyroscope variance (range 95.4–100%); voltage was used as fallback in 5 recordings where the gyro-reconstructed amplitude differed from voltage by > 20%. For the vertical (LARP/RALP) condition, chair velocity was further projected onto the head-transverse plane (× cos(70°), corresponding to the chair tilt-back angle that aligns the LARP/RALP canal planes with Earth-horizontal) to obtain the canal-axis-relevant angular velocity, then differentiated (d/dt) to yield the canal-projected angular acceleration used as the cross-spectral input. For the horizontal condition (head upright), the full chair angular velocity was differentiated to obtain the input acceleration.

#### 5.4.3. Velocity Step Pupil Analysis

Pupil area was aligned to acceleration onset and chair stop, baseline-normalized, and binned at 250 ms. Vertical-horizontal divergence onset was estimated from smoothed (1 s running mean) difference signals with bootstrap CIs (1000 iterations).

#### 5.4.4. Velocity-Referenced Gain Modeling

The vestibulo-pupillary pathway was modeled as a linear cascade of two physiologically motivated stages: cupula velocity transduction and iris smooth-muscle dynamics. The gain (magnitude) transfer function relating pupil area modulation to chair angular velocity is: H(jω) = (α / jω) / (1 + jω/ω_m), where α is the velocity gain, 1/(jω) represents cupula integration, and ω_m is the iris-muscle corner frequency. Because the absolute stimulus-frequency phase was not reliably resolved at this sample size, the model was fitted to the velocity-referenced gain alone and does not include a neural-delay term. Parameters were estimated by Nelder-Mead optimization with participant-level bootstrap CIs (3000 iterations).

The first stage, α/(jω), represents the cupula integrator. The semicircular canal cupula acts as an overdamped torsion pendulum whose dominant (long) time constant (~ 5–7 s in primates [6, 27]) exceeds the period of all stimuli used here (1.56–12.5 s); within this regime, cupula deflection is proportional to angular velocity rather than to angular acceleration [6, 27]. This velocity transduction is the defining characteristic distinguishing canal-mediated from otolith-mediated autonomic responses, as otolith organs encode linear acceleration. The scalar α (mm^2^ per deg/s) quantifies the overall sensitivity of the pathway from the vestibular velocity signal to pupil area change.

The second stage, 1/(1 + jω/ω_m), is a first-order low-pass filter representing the contractile dynamics of the iris smooth muscle. The corner frequency ω_m defines the bandwidth above which the muscle cannot follow neural drive; the corresponding time constant τ_m = 1/(2π f_m) reflects the mechanical relaxation time of the iris musculature, consistent with published dilator pupillae dynamics [24].

At low frequencies (ω << ω_m), the velocity-referenced gain is approximately flat, reflecting velocity coding; above the iris corner frequency the muscle low-pass progressively reduces the gain. The velocity-referenced gain is therefore determined by the two parameters (α, ω_m) without dependence on stimulus intensity. This linearity assumption is supported by prior characterizations of the VSR as a proportional, velocity-coded reflex [6, 27].

The two gain-model parameters (α, ω_m) were estimated by minimizing the sum of squared residuals (on a log scale) between the model-predicted and observed velocity-referenced gain at the three test frequencies (0.08, 0.16, and 0.64 Hz) using Nelder-Mead optimization. A separate acceleration-coded gain model (gain ∝ f, sharing the same iris low-pass) was fitted for comparison; the velocity-coded model fitted the gain approximately 53-fold better (ratio of summed squared residuals ≈ 53). Because two parameters are constrained by three gain points, the system is overdetermined by one and the fitted parameters are descriptive, requiring validation with additional test frequencies. The stimulus-frequency phase was not reliably resolved at this sample size and was not used in the fit. Confidence intervals were obtained by participant-level nonparametric bootstrap (3000 iterations): on each iteration, participants were resampled with replacement, the population-mean velocity-referenced gain was recomputed, and the velocity model was re-fitted; the 2.5th and 97.5th percentiles defined the 95% CIs (resampled fits retained when α ∈ [0.001, 100] mm^2^/(deg/s) and f_m ∈ [0.01, 50] Hz).

## Supplementary Files

This is a list of supplementary files associated with this preprint. Click to download.

- SupplementaryInformationfilev1.0.docx

## Figures and Tables

**Figure 1:**
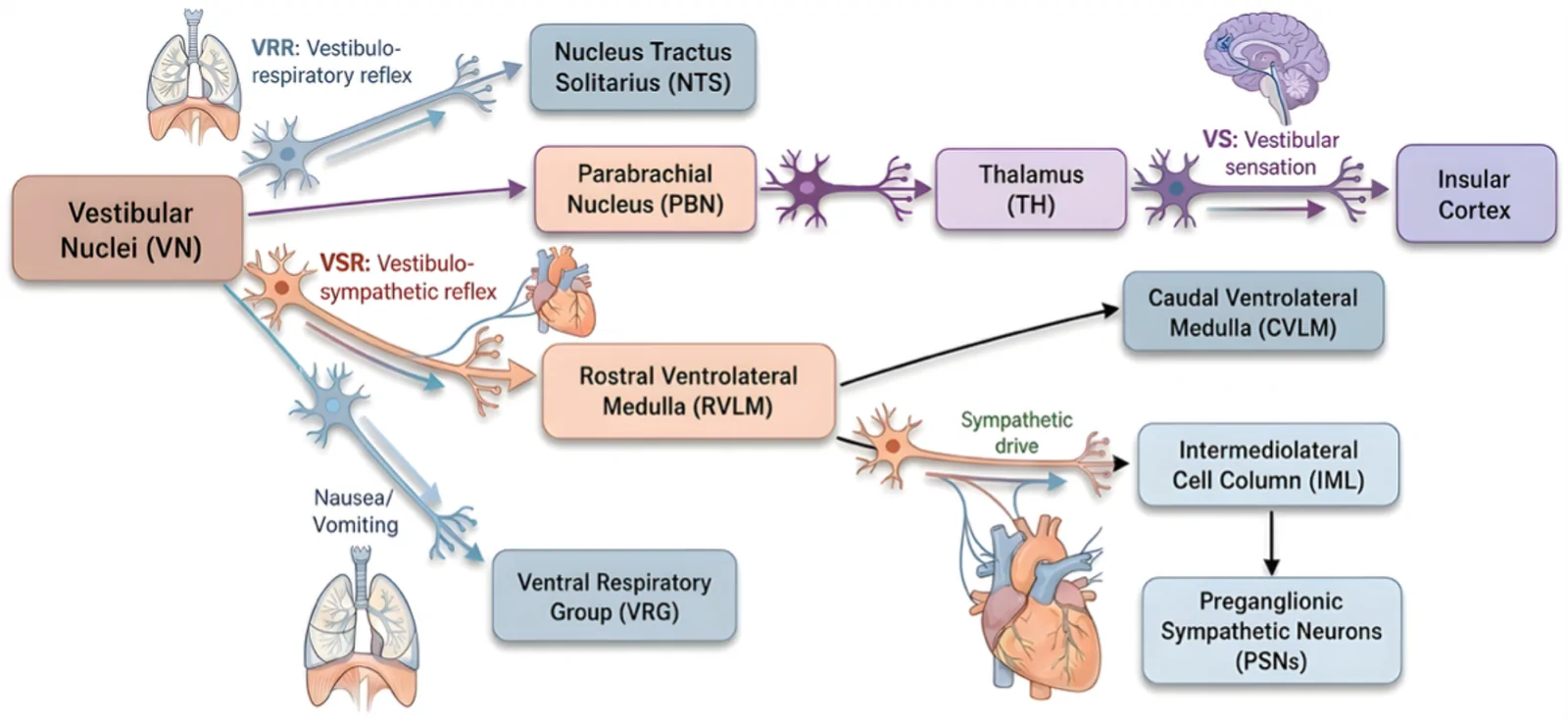
Schematic of vestibulo-autonomic neural pathways. The pathway from VN to RVLM constitutes the primary route for VSR. NTS mediates VRR, VRG contributes to nausea and vomiting, and the insular cortex is involved in VS [4–6]. VN: vestibular nuclei, RVLM: rostral ventrolateral medulla, CVLM: caudal ventrolateral medulla, IML: intermediolateral cell column, NTS: nucleus tractus solitarius, PBN: parabrachial nucleus, VRG: ventral respiratory group, TH: Thalamus, PSNs: preganglionic sympathetic neurons, VSR: vestibulo-sympathetic reflex, VRR: vestibulo-respiratory reflex, VS: vestibular sensation.

**Figure 2:**
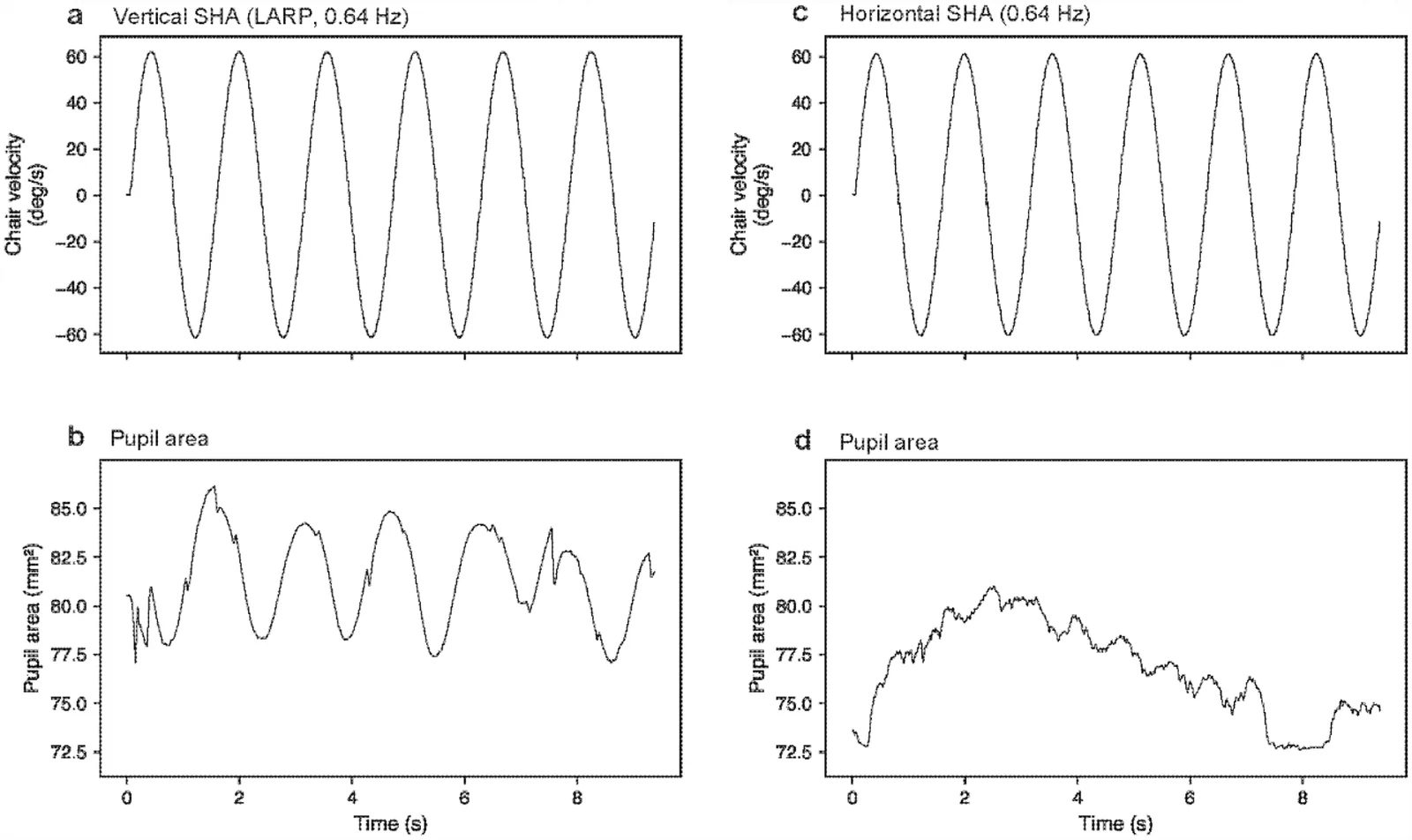
Stimulus-frequency–locked pupillary oscillation during vertical but not horizontal semicircular canal stimulation. Representative pupil-area recordings during sinusoidal harmonic acceleration at 0.64 Hz, selected by sinusoidal goodness-of-fit (R^2^) as illustrative examples of the vestibulo-pupillary response. (a) Chair angular velocity during vertical SHA (LARP canal plane) and (b) the corresponding pupil-area trace, which oscillates at the stimulus frequency. (c) Chair angular velocity during horizontal SHA at the same frequency and (d) the corresponding pupil-area trace, which shows no coherent oscillation at the stimulus frequency. Pupil-area panels (b, d) are plotted on a common y-axis. The presence of stimulus-frequency pupillary modulation during vertical but not horizontal rotation is consistent with a canal-plane–specific vestibulo-pupillary response; group-level quantification is provided in Figure 3 and Table 3.

**Figure 3:**
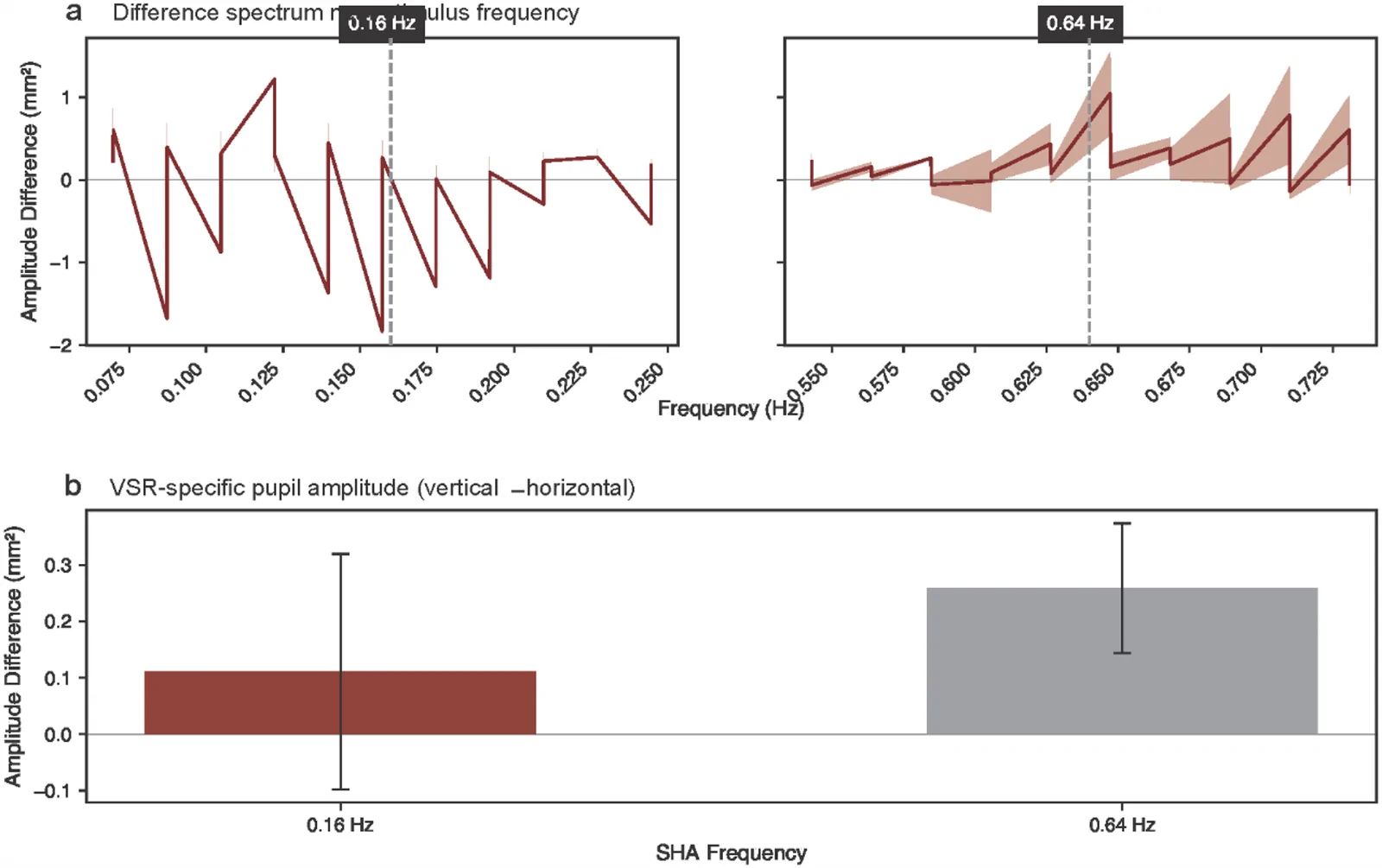
VSR-specific pupil signal isolated by spectral subtraction. Pupil area time series were decomposed into constituent frequencies using the fast Fourier transform (FFT), which quantifies oscillation amplitude at each frequency. The vertical-minus-horizontal difference removes non-specific components (arousal, hippus, measurement artifact), retaining only the canalplane-specific signal. (a) Difference spectrum zoomed to ±0.1 Hz around the stimulus frequency (dashed line). A positive peak at the stimulus frequency indicates greater pupil oscillation during vertical than horizontal SHA. (b) Mean ± SE amplitude difference at the stimulus frequency across participants. The displayed frequencies (0.16 and 0.64 Hz) show positive differences, and the vertical-minus-horizontal amplitude is positive at all three test frequencies (Table 3), indicating a VSR-driven pupil modulation specific to vertical canal stimulation.

**Figure 4:**
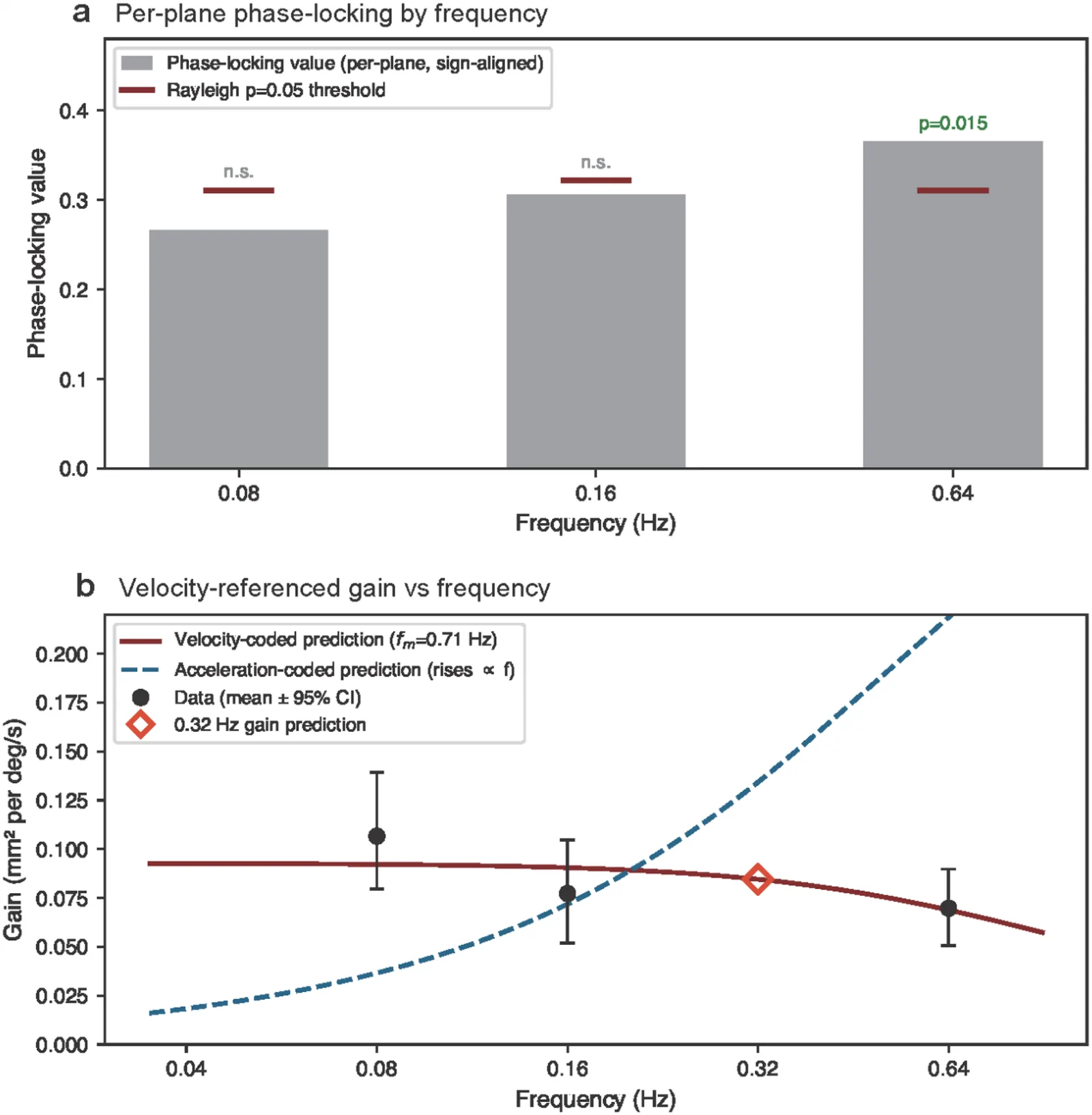
Vestibulo-pupillary dynamics: the pupillary oscillation is phase-locked to the stimulus, and the velocity-referenced gain is consistent with velocity coding. Gain model H(jω) = (α / jω) / (1 + jω/ω_m); the 1/f roll-off arises from cupula velocity integration. (a) Phase-locking value per canal plane (sign-aligned for the expected LARP/RALP antiphase) by frequency; the response is phase-locked, reaching significance at 0.64 Hz (Rayleigh p=0.015), with the two lower frequencies below threshold. The absolute phase – and thus a definitive velocity- versus acceleration-coding determination – was not resolved at this sample size. (b) Velocity-referenced gain (pupil oscillation amplitude per unit angular velocity) in the vertical condition at the three test frequencies (filled circles, mean ± 95% CI) follows the velocity-coded prediction (iris low-pass, f_m≈0.71 Hz) and diverges from the acceleration-coded prediction (which rises ∝ f); the velocity-coded model fits the gain ~53-fold better. The open diamond marks the a priori 0.32 Hz gain prediction (≈ 0.08 mm^2^/(deg/s) [0.06–0.11]).

**Table 1:** Mean change from baseline in autonomic metrics during vSHA (within-subject averaged, n = 18). W = Wilcoxon signed-rank statistic; r = rank-biserial correlation (small ≥ 0.1, medium ≥ 0.3, large ≥ 0.5). p-adjusted = BH-corrected across 15 tests. Blood pressure metrics showed medium-to-large effect sizes (r = 0.50–0.61) across all frequencies despite no individual comparison surviving correction (all p-adjusted > 0.05), consistent with a real but modestly powered vestibulo-sympathetic effect.

Metric	0.08 Hz Mean ± SD [W; p-value; p-adjusted; r]	0.16 Hz (Mean ± SD [W; p-value; p-adjusted; r])	0.64 Hz (Mean ± SD [W; p-value; p-adjusted; r])
SBP (mmHg)	5.77 ±11.43 [130; 0.055; 0.104; 0.52]	7.79 ± 12.49 [138; 0.024; 0.104; 0.61]	6.29 ± 15.25 [134; 0.037; 0.104; 0.57]
DBP (mmHg)	2.76 ± 5.42 [131; 0.050; 0.104; 0.53]	3.78 ± 5.94 [137; 0.026; 0.104; 0.60]	2.98 ± 6.98 [136; 0.031; 0.104; 0.58]
MAP (mmHg)	3.21 ±7.41 [128; 0.064; 0.107; 0.50]	4.51 ±8.09 [130; 0.053; 0.104; 0.53]	3.51 ±9.70 [136; 0.031; 0.104; 0.58]
HR (bpm)	0.92 ± 4.77 [95; 0.394; 0.537; 0.11]	2.03 ± 4.97 [124; 0.098; 0.147; 0.45]	0.11 ±6.62 [89; 0.896; 0.896; 0.04]
RR (breaths/min)	0.03 ± 2.68 [79; 0.794; 0.851; −0.08]	0.31 ±2.82 [94; 0.711; 0.851; 0.11]	0.03 ± 2.62 [83; 0.776; 0.851; −0.03]

**Table 2:** Mean change from baseline in autonomic metrics during vVST (within-subject averaged).

Metric	Combined (n = 17) [W; p_raw; p_adj; r]	LARP (n = 16) [W; p_raw; p_adj; r]	RALP (n = 16) [W; p_raw; p_adj; r]
SBP (mmHg)	8.74 +/− 11.38 [127; 0.018; 0.053; 0.66]	7.69 +/− 10.45 [114; 0.019; 0.053; 0.68]	9.88 +/− 15.66 [112; 0.023; 0.053; 0.65]
DBP (mmHg)	4.50 +/− 5.95 [124; 0.024; 0.053; 0.63]	4.06 +/− 5.38 [114; 0.017; 0.053; 0.68]	5.00 +/− 8.16 [112; 0.024; 0.053; 0.65]
MAP (mmHg)	5.24 +/− 7.70 [124; 0.025; 0.053; 0.63]	4.38 +/− 6.98 [110; 0.032; 0.053; 0.62]	6.19 +/− 10.58 [110; 0.032; 0.053; 0.62]
HR (bpm)	2.26 +/− 5.23 [111; 0.107; 0.146; 0.45]	2.38 +/− 5.06 [90; 0.088; 0.132; 0.33]	2.06 +/− 6.06 [80; 0.255; 0.318; 0.18]
RR (breaths/min)	−0.24 +/− 2.76 [60; 1.000; 1.000; −0.22]	0.12 +/− 2.33 [42; 0.812; 0.870; −0.38]	−0.38 +/− 2.87 [46; 0.680; 0.785; −0.33]

W = Wilcoxon signed-rank statistic; r = rank-biserial correlation (small ≥ 0.1, medium ≥ 0.3, large ≥ 0.5). p-adjusted = BH-corrected across 15 tests. Blood pressure metrics showed large effect sizes (r = 0.62–0.68) across all positions, with corrected p-values approaching significance (all p-adjusted = 0.053).

**Table 3:** Stimulus-frequency pupil amplitude, velocity-referenced gain, and cross-plane phase-locking during SHA. Values are per-condition means across participants. Amplitude = stimulus-frequency pupil oscillation amplitude (single-sided FFT); velocity-referenced gain = pupil-to-velocity amplitude ratio. Cross-plane PLV = sign-aligned phase-locking value of the pupil across the LARP and RALP canal planes (defined for the vertical condition only; the horizontal condition has no canal-plane pair), with the Rayleigh test result in parentheses; the response reached significance at 0.64 Hz (Fig. 4b). N varies (16–18) due to exclusion of trials with < 50% valid pupil samples after blink removal. Vertical amplitude and velocity-referenced gain exceeded horizontal at all three test frequencies.

Condition	Freq (Hz)	N	Amplitude (mm^2^)	Velocity-ref. gain (mm^2^/(deg/sj)	Cross-plane PLV (Rayleigh p)
Vertical	*0.08*	17	2.17	0.107	0.27 (n.s.)
Vertical	*0.16*	16	1.56	0.077	0.31 (n.s.)
Vertical	*0.64*	17	1.37	0.070	0.37 (p = 0.015)
Horizontal	*0.08*	18	1.51	0.025	—
Horizontal	*0.16*	18	1.07	0.018	—
Horizontal	*0.64*	18	1.02	0.017	—

**Table 4:** Velocity-referenced gain model parameters.

Parameter	Estimate	95% CI	Interpretation
α (mm^2^/(deg/s))	≈ 0.09	[0.07, 0.12]	Pupil modulation per unit angular velocity (velocity-referenced gain; velocity coding favored over an acceleration-coded drive)
f_m (Hz)	≈ 0.71	[0.45, 1.8]	Iris smooth muscle corner frequency

Estimated from H(jω) = (α / jω) / (1 + jω/ω_m). The two-parameter gain model (α, f_m) was fitted to the velocity-referenced gain at three frequencies; estimates are descriptive and require validation with additional frequencies. 95% CIs from participant-level bootstrap (3000 iterations). α = velocity-referenced gain (overall pathway sensitivity); f_m = iris smooth-muscle corner frequency (above f_m, the muscle cannot follow neural drive). The velocity-coded model fitted the gain approximately 53-fold better than an acceleration-coded alternative; the model contains no neural-delay term, and the absolute phase was not resolved at this sample size.

## Data Availability

The datasets generated and/or analysed during the current study are not publicly available due to participant privacy and institutional ethical restrictions but are available from the corresponding author on reasonable request.
